# Causal models and causal modelling in obesity: foundations, methods and evidence

**DOI:** 10.1098/rstb.2022.0227

**Published:** 2023-10-23

**Authors:** Roger S. Zoh, Xiaoxin Yu, Philip Dawid, George Davey Smith, Stephen J. French, David B. Allison

**Affiliations:** ^1^ Department of Epidemiology and Biostatistics, Indiana University School of Public Health-Bloomington, Bloomington, IN, 47405-7000, USA; ^2^ University of Cambridge, Cambridge, CW3 OWB, UK; ^3^ MRC Integrative Epidemiology Unit (IEU), Bristol Medical School, University of Bristol, Bristol, UK

**Keywords:** causation, counterfactual, randomization, causal Inference, causal model, assignment mechanism

## Abstract

*Discussing causes in science*, if we are to do so in a way that is sensible, begins at the root. All too often, we jump to discussing specific postulated causes but do not first consider what we mean by, for example, *causes* of obesity or how we discern whether something is a cause. In this paper, we address what we mean by a cause, discuss what might and might not constitute a reasonable causal model in the abstract, speculate about what the causal structure of obesity might be like overall and the types of things we should be looking for, and finally, delve into methods for evaluating postulated causes and estimating causal effects. We offer the view that different meanings of the concept of causal factors in obesity research are regularly being conflated, leading to confusion, unclear thinking and sometimes nonsense. We emphasize the idea of different kinds of studies for evaluating various aspects of causal effects and discuss experimental methods, assumptions and evaluations. We use analogies from other areas of research to express the plausibility that only inelegant solutions will be truly informative. Finally, we offer comments on some specific postulated causal factors.

This article is part of a discussion meeting issue ‘Causes of obesity: theories, conjectures and evidence (Part II)’.

## 1. Introduction

We are going to talk about cause and its variations, such as causal effects, causal models and causal inference. The concept of causation, its ontological status and methods for evaluating causal effects can be fraught and the subject of differences of opinions, even among thoughtful scholars of the topic. Indeed, even the current authors enjoy debating these questions, and the following text represents a concatenation and not necessarily a consensus of our views. For the purpose of this paper, we assume that causes are, in principle, knowable. However, not all philosophers of science accept these assumptions as self-evident, including a member of the Royal Society, Bertrand Russell (1872–1970). Almost exactly 100 years ago, Russell said this about cause and effect at a presentation at the Royal Society: ‘It's not something we know exists. It's a way of thinking about things’. As scientists, we think about propositions and questions, try to collect evidence as objectively as possible, review those bits of evidence collected and then update our beliefs. That sequence implies that one must start by saying one does not know everything, so one starts with ignorance and confusion. As good scientists, we accept that we are ignorant and confused, but then we work on becoming less so. We start with some premises and observations, frame hypotheses and questions, design studies, and so on. In the words of Richard Feynman, ‘The first principle is that you must not fool yourself—and you are the easiest person to fool’ [1]; therefore, as scientists, we must ensure that we are clear and critical in our approaches to the design and interpretation of studies and to what constitutes cause.

Too often, our thinking about causes is cluttered with glossing, smoothing over, or transitioning from one thing to another without much clarity in the approach. For example, when thinking about the cause of obesity, are we asking what the cause of the secular increase in obesity is? Does *this* cause the obesity pandemic? Could *this* have contributed to the obesity epidemic? Equivalently, research findings are often presented as ‘in the population, people who do more of *this* have less of *that*’ or ‘this gene seems to be associated with that trait/phenotype within the population’. Nevertheless, the question remains: why does one person (or some people) become obese and others remain lean? Also, do those questions tell us necessarily what caused the obesity pandemic or the secular increase in obesity or what change occurs when people consume a particular diet or take a specific drug or treatment? There are yet other questions one could ask.

Our paper is structured as follows: Section 2 discusses the difference between mathematical equations and causal models. Section 3 discusses some linguistic issues pervasive in obesity research. We provide some guidance on what constitutes a reasonable causal model in §4. Section 5 attempts to catalogue methods and issues of estimation in causal models. Section 6 discusses the plausibility that only inelegant models will suffice. We close with some concluding remarks in §7.

## 2. There is no formulaic scientific method

As we consider the study of causal effects and causal theories in obesity-related research, let us ask about some fundamentals of causation itself as well as about scientific approaches to studying causation. With respect to causation itself, some scholars have noted that it is the intellectual wellspring from which we all drink. That is, we are motivated to estimate causal effects, determine whether causation exists and to find causal outcomes. The thought is that in doing so, one not only comes to understand the world in a meaningful way but also learns which manipulations can achieve desired ends.

And yet, philosophers of science have noted that whether causation even exists is not clear. If one considers atoms and molecules and the cells, tissues, organs, bodies and other conglomerations that atoms and molecules compose, beyond the Cartesian doubt expressed in the phrase ‘Cogito ergo sum’, we can take their existence as not in question. We can weigh them, measure their volume, observe some of them under a microscope, excise some and hold some in our hands. But we cannot hold causation in our hands. We cannot observe causation under a microscope or excise it or weigh it or measure its volume. ‘Where is causation?’ Causation is a concept involving the relations among physical elements of the world. Does it exist only in our minds, or is there some reality to it? Must it obey certain rules, such as those often expressed in temporality? Here at the Royal Society, this was questioned long ago by Bertrand Russell. And to this date, philosophers of science still struggle with and debate these questions [2–4]. We will not try to solve these here but rather say that for the purposes of this paper, as for the whole conference on which it is based, we assume that causation is meaningful and does, in some sense, exist. We assume that causation is, in principle, discernible and can be tested via methods, at least in some cases.

What are those methods? Clearly, one might say they should be some subset of ‘the scientific method’. And yet, once again, despite some platitudinous introductory writings to the contrary, most current methodologists and philosophers of science do not believe there is any such thing as ‘the’ scientific method. This is laid out clearly by scholars such as Henry Bauer, Stuart Firestein, and Lee McIntyre [5–7]. Although there may not be a defined set of procedures that give a recipe-like way to enact ‘the’ scientific method, scientists regularly use a few elements in conducting research that generally merit the appellation ‘scientific’.

We begin by identifying premises or observations on which to build questions or hypotheses. From there, we frame questions or hypotheses to be addressed. Then, we design studies to answer or test these questions or hypotheses. We then implement and execute these studies. The data or observations that we collect are then analysed. Finally, we interpret and communicate the results.

## 3. Linguistic issues: what do we mean by *causes* of obesity?

Just as it is valuable to define what we mean by *cause* (which the authors would consider in the context of an experimental situation is an unarguable link between an intervention and an observed outcome), it will be valuable to consider other uses of language involving cause in the field of obesity that may not always be defined well, if at all. For example, many adjectives are placed before the word *cause* presumably to imply something, yet it is not clear to us what is necessarily implied or whether there is an explicit definition of what is implied. The text box 1 includes some quotations from the literature illustrating the use of such terms.

Box 1. Major cause: ‘In this context, current epidemiological trends in weight for height measurements indicate that a major cause of the global obesity problem lies in dietary and physical activity patterns, while genetic and metabolic studies reveal that there are individuals who are more susceptible to weight gain than others' [8].

Primary cause: ‘Studies identified that examined energy intake and energy expenditure were then placed into one of three categories (cross-sectional with nationally representative sample, cross-sectional among population subgroups and longitudinal studies). The authors concluded that there is inconclusive evidence for the primary cause of obesity in children’ [9].

Better explanation: ‘The impact of genetics as a prerequisite over other factors, e.g. sociological or physiological, is yet to be determined. Because of the rapid increase in obesity within the past 30 years, it is difficult to predict to what degree genetics plays a role in a person becoming overweight or obese. This rapid increase throughout various populations suggests behavioural, lifestyle, and cultural changes are better explanations for the increase in obesity’ [10].

Principal cause: ‘The problems of overweight, obesity and obesity linked declination in immune response, are not new to society but they have become more complicated nowadays. The principle [sic] causes of these problems are high fat diet, lack of healthy diet, excessive eating and lack of physical exercise’ [11].

More important cause: ‘On two of the cause factors, ‘modern technology and media’ and ‘physical activity environment’, women had significantly higher scores than men, indicating that they considered these to be more important causes of childhood obesity’ [12].

Predominant cause: ‘Obesity cardiomyopathy typically occurs in persons with severe and long-standing obesity. The predominant causes of death in those with obesity cardiomyopathy are progressive congestive heart failure and sudden cardiac death’[13].

We do not know what terms like ‘primary cause’ or ‘principal cause’ mean precisely. We suggest that authors do not use or define terms like these clearly or explicitly. How then can we evaluate whether, in fact, any research questions or hypotheses involving those terms have been answered or answered adequately?

Accepting that there are causes and that some causal effects are not zero, then causes presumably exist in some quantity. As E. L. Thorndike famously wrote, ‘All that exists, exists in some amount and can be measured’ [14]. These linguistic modifiers seem to imply a quantity of causation, but how we quantify causal effects in a way that is useful and meaningful?

There is no single way to quantify causal effects, especially when we may change the outcome variable or its scaling for which we wish to estimate an effect of some cause or causal factor. Many measures of effect size exist [15]. Some, like percent or proportion of variance explained (i.e. the proportion of variation due to some independent variable in a causal regression-type model relative to the total variance in the outcome), scale things in terms of relative variances. This is one useful and popular method that seems especially common when describing the purported causal effects of genetic variants on traits in human populations. While this method is entirely reasonable, it is notable that such values depend not only on the effect of a particular allele or genotype on some aspect of the location of distribution (e.g. the mean most often, but other location parameters^1^ such as median or other quantiles could, in principle, be used) but expresses this in terms of variances. Such proportions of variance are, therefore, highly dependent upon the variability of the causal factor (in this case, genotype) in question. Such metrics, therefore, do not tell us what the causal effect ***could be*** but rather what the causal effect on variability ***is now*** under current circumstances. This is notable because some authors may dismiss small percentages of variance in a particular circumstance that prevails in the environment as indicating that the causal factor is unimportant. However, the causal factor might be very important if it is manipulable, which would then alter its variance in the population. Consider arguendo the variance due to a drug that has not yet been developed. Before the drug is developed and introduced into the environment, there is no variability in taking the drug, and therefore it can contribute no variance to an outcome. But that does not mean that the development of that drug would be trivial. Similarly, precious little variation in the outcome of diabetes in the general human population is due to genetic variants in the insulin gene [16], and yet few would argue that insulin is not an important variable in understanding and treating diabetes.

## 4. What constitutes a reasonable causal model?

### (a) Not all so-called models are causal models

If we consider a model as a representation of a scientific system (or part of a system) that we can test to provide evidence to support or refute a hypothesis, linguistic issues pertain to how we conceive of any causal model and whether our verbal descriptions are sensible. Just because someone has articulated grammatically correct phrasing in question format does not mean that they have asked a meaningful question. Consider the following question: ‘at what temperature does the number seven melt?’ [17]. This is a grammatically correct question, but it has no meaning. Equivalently, someone referring to the 'XYZ model' does not mean that the thing referred to is not *an* (rather than *the*) XYZ model and that other things could not also be called the XYZ model. Or, because somebody asks about the XYZ model, does this mean there *is* an XYZ model? With some of the models we are being asked to compare in obesity research, it is important to ask: is there a model? Because, in some cases, the very existence of the named model may not be established.

Another linguistic issue is related to the formulation of a causal model. Some causal models are expressible in equations. Yet, not all equations are causal models. This is an equation:

Energy Stored=Energy In – Energy Out.

We accept that this equation is true if we accept the law of conservation, which we are not prepared to dispute. Some might say we can rearrange this equation and ask in which direction the causation flows, or one can find published papers that ask about the direction of causation, and ask ‘Can we reverse the causation?’ Because this is an equation, we can rewrite it by moving terms to different sides by using subtraction or addition. We can ask in which direction the causation flows. But why even assume there is a direction of causation? Just because it's an equation doesn't mean it's a model of causation. There may be no model.

**Figure 1.**
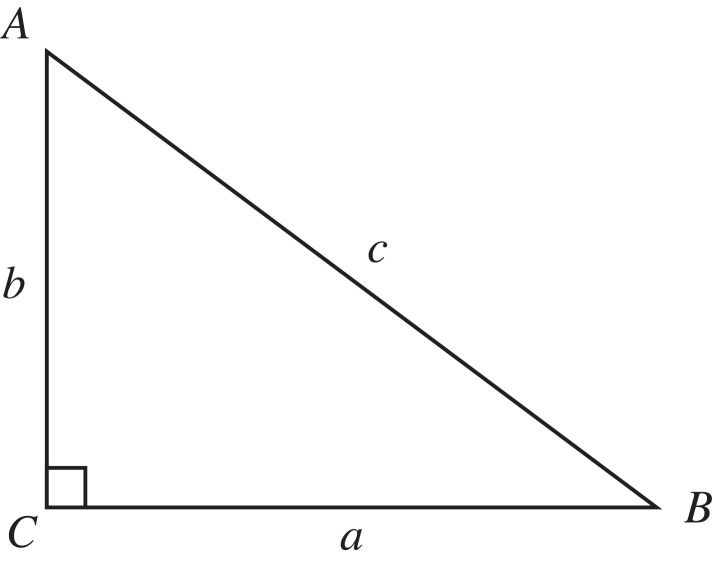
Right triangle.

Let's consider another equation with three terms related by addition: the Pythagorean theorem in the right triangle. Consider the following right triangle ABC in figure 1.

Suppose one claims that ‘When we make the legs of the triangle ABC bigger, namely *a* and *b* are bigger, then the length of the hypotenuse, denoted by *c*, gets bigger’. This statement, albeit grammatically correct, is unsettling. You can think of a bigger triangle, but the triangle does not get bigger. The hypotenuse does not get bigger because the legs are bigger, and the legs do not get bigger because the hypotenuse gets bigger. When you say that the hypotenuse is *bigger*, you have essentially said that the legs are bigger because that is inherent in the right triangle definition. There is no causal model here to ask about which direction causation flows. There's no model.

### (b) From the heavenly platonic plane to the earthly diet debates

What has been sometimes referred to as the energy balance model, simply by pointing to the equation above, is really nothing more than a constraint on how all models must behave if we are to accept the law of conservation. That is, this is not a causal model to be refuted or confirmed but rather it is a constraint under which other models must operate. Thus, it is not in any way incompatible with models in which there are causal effects of elements of dietary consumption other than energy intake on obesity levels. This is so for many reasons. First, the constraint as described in the equation above refers to components of energy, including total body energy stores. One should not conflate total body energy stores with body fat. Under some circumstances, they are highly correlated, but they are not the same. That lack of sameness is part of the issue. The composition of the diet, independent of energy intake or how the diet is consumed (e.g. the time of day it is consumed, whether it is eaten hot or cold) could all affect nutrient partitioning, where by *nutrient partitioning* we mean in what tissues or bodily components any body energy is stored. For the same amount of stored body energy or increased stored body energy, a greater or lesser amount could be stored in body fat depending on other factors, which may include the composition of the diet or the way in which the diet is consumed. This therefore allows the possibility that elements of diet composition, including but not limited to the proportion of the energy intake that is carbohydrate, can affect body fatness without in any way violating the energy balance equation (i.e. the constraint under which all causal models must be considered to hold if we are to respect the ideas of *consilience* as described below).

Additionally, the constraint or energy balance equation portrayed above does not include a time element. If we reexpressed the equation to involve delta energy in minus delta energy out equals delta energy stores, where delta implies change over some period of time, then we have a richer conception. Now, we can ask how composition of the diet consumed—independent of energy consumed, or the manner in which the diet is consumed—could affect subsequent elements of energy balance. For example, eating a diet of one particular composition might lead to greater consumption of energy at subsequent points in time or lesser consumption of energy at subsequent points in time. So too could eating a diet of different composition or eating a diet in a different manner—independent of the energy content of the diet at that interval in time—lead to different degrees of energy expenditure or even energy excretion or losses in faeces and urine. Let us consider a carbohydrate–insulin model, which proposes that a high-carbohydrate diet, comprising refined starches and sugars, elevates insulin secretion that in turn directs the partitioning of energy toward storage as fat in adipose tissue and away from oxidation by metabolically active tissues [18]. There is nothing about this model as requiring competition with the energy balance equation (not model of causation), nor is there anything unique or especially novel about the so-called carbohydrate–insulin model in specifying that something other than the energy intake of the diet at some points in time can affect subsequent adiposity independent of the total energy consumed in that diet at those points in time. Any idea that there is some sort of inherent conflict between the law of conservation and some model involving carbohydrates or other dietary composition seems on an equal logical footing with asking at which temperature the number 7 melts. Readers interested in reading more about the carbohydrate–insulin model and the debate that it has generated in the field of obesity are directed to the following references [19,20].

### (c) Model of obesity

We speak much of causation and causal effects in obesity-related research. However, researchers need to think more clearly about what their research is actually measuring and be more precise in the language used to describe the outcomes. Here, we briefly offer a few thoughts aimed at the linguistic and conceptual levels to contribute constructively to the dialogue around causal effects in obesity research.

First, it is essential to recognize that there are often unspoken premises in many obesity research questions or statements.

1. When a person says they are addressing *the* X model of obesity, it implies that there is a thing called the X model of obesity that the scientific community, in general, understands (or should understand) to mean the model that the author is putting forth. This is in contrast with the author putting forth the idea that this is *an* X model of obesity (as opposed to the X model of obesity). This would allow the implication that there are other ways to conceive this model and that perhaps other parties involved in the dialogue or intellectual opponents in consideration of various models might in fact not accept that the model put forth does indeed merit its appellation as *the* X model of obesity.
2. When someone states that they are studying the X model of obesity, it is not clear what this is a model of. To say it is modelling obesity does not make clear whether it is modelling the causal effect of X on weight change in individuals over time, the cause of the obesity pandemic or more generally secular changes in obesity, the degree to which the purportedly independent or ‘exogenous’ variables in the X model have contributed to variance in individuals' adiposity levels within the population (which is highly dependent not only on there being causal effects of those variables but on those variables having sufficient variability within the population for those effects to be palpable), or what those effects could be in the environment. By analogy, consider the impact of COVID-19 vaccines in early 2020. If we ask what the effects of such vaccines were on the risk of getting COVID-19, the temptation might be to say zero. From one point of view, this is true because there were no such vaccines. But now that vaccines are available, that answer would be incorrect. So it behoves us to make clear whether we are talking about the causes of the pandemic of obesity, the causes of an individual's obesity, the causal factors contributing to individual differences among persons within the population, or the causal effects of factors on obesity or obesity indicators such as adiposity. These may all be of interest, but they are fundamentally different and are often conflated.
3. We succumb to many flaws in logical reasoning. In the context of thinking about causation, one flaw is the notion that analogy proves something without ancillary assumptions. Therefore, if we wish to state that A is a cause of B and we point to something similar to A (e.g. A′) and say ‘A’ is a cause of B; hence, we can conclude that A is a cause of B″, this argument is unreasonable unless one relies on implicit ancillary assumptions that all parties understand and on a legitimate basis accept to be true. If there are some characteristics of A′ that are sufficient to make it a cause of B, and if it is well known (and justifiably well known) to all that A has those very same characteristics and no other characteristics that would negate them, then the analogy is fair. Otherwise, it is at best insufficient to carry the argument as a demonstration that A is a cause of B. For example, Dhurandhar^2^ argues that lifestyle choices cannot be considered causes of obesity ***because*** oedema is not due to excess water consumption. This argument does not seem sound. For it to be so, we would need to know that the statement about oedema is correct *AND* that the analogy between the fluid balance case and the obesity case has all the right characteristics in common and no nullifying characteristics differently.

So let us ask questions. Let us ask, what exactly do you mean by that? Let us ask, what exactly is the causal effect you are trying to comprehend? Let us ask, what is your justification for saying that this thing you are calling *the* X causal model of obesity is not rather *an* X causal model of obesity? Is the model as you depict it the same as the model as others would depict it? Is the model as you depict it the model as the general scientific community would understand it or is it just one instantiation of many possible models that might receive that label? Let us ask, is it a causal model at all?

### (d) Models involve assumptions

Like any statistical model, a causal model is an attempt to describe a real-world situation. For it to be useful, we must understand:

1. its *syntax*: how it behaves and can be manipulated mathematically;
2. its *semantics*: how terms in the model relate to the real world being modelled;
3. its inbuilt *assumptions*: what (using 1 and 2) it says about how the real world behaves.

When the assumptions are believable, then using semantics, we can translate a real-world query into a model statement, manipulate that syntactically to produce an answer (or show that this is not possible) and translate that answer back to the real world, thus allowing inferences to be drawn. A model will usually involve unspecified parameters, which can be informed by data.

When the situation being modelled is an experiment, we might get by with a very simple/parsimonious statistical model, as the experimental approach attempts to control the environment in which the treatment variables are the ‘only’ changing variable (which of course is not necessarily the case). Thus, we might model the response Y of a unit receiving treatment (denoted by t) as normal with mean depending on t, and common variance, all responses being independent. The use of data to check this model and infer its parameters is a standard focus of elementary statistics.

More complex causal modelling is required for observational situations in which multiple variables may be different between situations under observation. A query might be of the form, ‘What would happen if we intervened in the system according to option one versus option two?’ [21]. Then the model also needs to relate the observed system with a perturbed but unobserved system—this relationship again being subject to the above three criteria.

Currently popular causal frameworks pay some attention to syntax (on which they differ), less to semantics, and rather little to assumptions. Consider the simplest example of a causal variable, denoted in the model by X (diet intervention or medication) and a response variable, denoted by Y (weight loss). Any approach needs to clarify the real-world understanding of model ingredients such as probability (because we are dealing with probabilistic models), of the specific variables X and Y, and, especially, of an intervention in X.

The *potential outcome* framework [22] involves syntactical terms such as Y_x_, interpreted as the value that Y would take, were an intervention to set X to x.^3^ It is typically assumed (consistency) that X = x implies Y = Y_X_ when both X and Y arise in the observational setting, so connecting the two settings.

The *decision-theoretic* framework [24] involves conditional distributions P(y | x), relevant to the observational setting, and interventional distributions P(y | do(x)) = P(y_x_)_,_ interpreted as the probability distribution of Y, following an intervention to set X to x. These need not be the same but might be assumed related in various ways, perhaps involving further variables.

A *structural causal model* [4], like a *structural equation model*, invokes an unobserved random ‘error variable’, E, and expresses Y as a deterministic function Y = f(X, E). Neither E nor f needs to have any real-world counterpart. Both the distribution of E and the function f are implicitly supposed to be the same, whether X arises naturally (as in the observational situation) or by intervention, again relating the settings.

When more variables are involved, it can be useful to represent and manipulate the model by means of a graph, often a directed acyclic graph. This can be done in any of the above frameworks (though this is rare for potential outcomes). In each case, additional syntactical relationships should be specified between the basic model terms (e.g. variable, intervention) and aspects (e.g. node, arrow) of the graphical representation. However, these are often left unspecified. Graphical representations without clear syntax and semantics can be problematic [25].

## 5. Methods and estimation of causal effects

### (a) Issues in non-randomized studies

#### (i) General confounding^4^ and other biases

The idea that rigour in the execution of a non-randomized study is insufficient to confidently eliminate other threats through inferences of causation is not mere theoretical speculation. This is not to say that no set of non-randomized observations can ever permit reasonable inferences of causation. Indeed, many astute authors have written about when and how it is reasonable to try to make causal inferences from non-randomized data [21,27]. However, that does not mean that these will be effective in many cases, especially those in which the only alternative study design used is an ‘ordinary association test (OAT)’, as described by Brown and colleagues [28].

This was put to the test in an illustrative case creatively by Ejima *et al.* [29]. They showed that in a mouse study designed such that it contained both a randomized element and an association element, each of which could look at the association of energy intake with longevity in mice (in the experimental condition indicating a causal effect and in the non-experimental aspect indicating an association but not necessarily causation), interesting outcomes were observed. Notably, the mouse study was far more rigorous than any human observational epidemiologic study could ever realistically be. The mouse was isogenic, using in-breeding. Therefore, there was no genetic variation to confound anything. The mice were fed the same composition of food, did not smoke, did not drink alcohol, were prohibited from mating, were raised in the same kinds of housing (cages), had the same light–dark cycles, etc. And yet, completely opposite findings were observed in the association element versus the randomized experimental element. In the randomized experimental element, the causal effect of being assigned to consume less energy was a longer lifespan. In contrast, the association between energy consumed and lifespan was positive in the association element, meaning that animals that spontaneously chose to eat more lived longer. This was likely due to general health being associated with greater appetite and longevity. Yet, stating that this explanation is likely the case does not take away from the fact that this illustrative example shows that even the most rigorously designed observational study will not necessarily recapitulate the causal effect that would be obtained in a randomized experiment.

#### (ii) No amount of rigour will overcome the problem completely

While randomized experimentation is the gold standard for estimating causal effects, randomized experiments are often impossible to conduct for many reasons: practical, ethical, financial, etc. Then recourse may be had to purely observational data. However, observational studies raise several inferential challenges, the most important being the high likelihood of confounding. Consider, for example, comparing recovery rates under two different treatments. Confounding can occur when we are not comparing like with like because of pre-existing differences between the treatment groups. For example, suppose that perhaps, unknown to the analyst, treatment 1 were given preferentially to individuals predicted to recover and treatment 2 to individuals not so predicted. Then the responses would look different between the treatment groups, even if the treatments were in fact, identical and a face-value analysis of the data would be misleading.

In such a case, the probability of survival for an individual *observed* to have been given a particular treatment (and so having been predicted to survive) would not be the same as the probability of survival for a random individual, not so observed, who was *assigned* to that treatment in an experimental setting. This difference between the observational and experimental settings is seen by some as the essence of confounding.

A typical ploy to handle confounding is to condition on (control for) further information about an individual. For example, if we knew which patients were predicted to recover, comparing the two treatments within this group might be treated as not further confounded, hence causal. Or, if age and sex were thought to affect the choice of treatment as well as the outcome, the coefficient of treatment, in a regression of outcome on treatment, age and sex, might be imbued with causal meaning. Ignoring such covariates can induce confounding through ‘omitted variable bias’. However, a set of ‘sufficient covariates’—variables existing prior to treatment, conditioning on which is sufficient to eliminate confounding—cannot be identified from observational data alone because this property involves a comparison with what would happen in an experiment. It is, therefore, incumbent on an analyst to give, and to be prepared to defend, reasoned arguments for their chosen covariates. Unobserved covariates that affect treatment choice and outcome are called *confounders*, but this property alone is insufficient to render them sufficient covariates (and hence unconfounders) when observed. Confounding can, to a certain extent, be offset by effect size, whereby if the effect size is sufficiently large in comparison to other variation between groups (or in comparison to similar studies investigating other treatments in similar populations, diseases, etc.), that other possible confounding biases cannot be thought to be responsible. For example, few would argue that cigarette smoking causes lung cancer, as the association is so large that alternative confounders cannot be imagined to be responsible.

It is important that we only allow for pre-treatment variables. Conditioning on other variables can induce bias where it would otherwise be absent [30]. *Post-treatment bias* can occur when we mistakenly allow for a *mediator* variable: one that is affected by treatment and in turn, affects the outcome. Even in an experimental setting where the outcome depends on the treatment, conditioning on a mediator can mask this dependence*. Collider bias* can occur when we mistakenly condition on a variable that is affected by both treatment and outcome. Other biases are still more subtle. *M-bias* [31] can occur when we have a pair of jointly sufficient covariates but we condition on a pre-treatment variable that is affected by both, which can destroy sufficiency.

Relationships between the variables in a problem are often represented by means of a *causal diagram* [4]: a directed acyclic graph whose arrows encode direct dependence. This can then be interrogated to identify sufficient sets of covariates, which need not be unique. But note that such a graph already embodies sufficiency assumptions—in particular, the parents (this refers to ‘parent variables' as defined by Pearl and not [necessarily] to actual human parents) of any variable are supposed to be sufficient to control for confounding for their child—and these assumptions still require external justification, including judgement of plausibility.

### (b) Principles and goals of randomization

#### (i) Randomization

It is not enough to be aware of randomized experiments and other study designs; we must implement them properly, understand their effects and interpret them appropriately. Randomization occupies a critical role in causal inference [32]. However, we know that in the field of obesity, as Vorland *et al*. [33] have shown, many errors are published in the actual implementation of what is called random assignment such that what is called random assignment in the literature often is either not random assignment or is random assignment somehow misapplied. This threatens the causal inferential strength of nutrition and obesity research. Second, as Owora *et al*. [34] have articulated, many confused statements in the literature indicate that many investigators do not understand how randomization works at a conceptual level and what randomization does and does not guarantee. Therefore, increasing the fidelity of interpretation at a fundamental conceptual level is important. Notably, more clarity is warranted between randomized experiments as a process versus the outcome of randomizing because many investigators falsely think that randomization can fail, leading to a biased study. In the general terminology of frequentist statistics, outcomes of single studies are neither biased nor unbiased (though they may be erroneous or not). However, the process of conducting the study by which the outcome was produced may be biased or unbiased. Bias refers to the process, not the outcome. Randomization, if adequately conducted as a process, cannot fail^5^.

Finally, particularly in the childhood obesity literature, it has been noted that there are many problems in the interpretation of intervention effects and study designs, as noted by Brown *et al*. [35] and summarized further in a video from New Zealand [36] and a *New York Times* commentary [37]. Clearly, more training is merited on this.

#### (ii) Cluster randomized trials

Finally, cluster randomized trials are becoming increasingly used in the obesity and nutrition research in general and in childhood obesity and nutrition research in particular. Cluster randomized controlled trials (cRCTs) are multilevel experiments in which groups of study participants who are not constituted at random (e.g. schools or workplaces) are randomly assigned to the experimental conditions, and all study participants within that group (i.e. cluster) are intended to receive the treatment to which their cluster is allocated [38]. Yet, these trials often seem so poorly designed, misanalysed or misinterpreted as to be truly remarkable. Some of the current authors have commented on this in many papers [39–42] and have had many papers corrected or retracted [33,38,43]. As things currently stand, consumers of research who read about the causal effects of treatments in the childhood obesity literature, including cluster randomized trials, must frequently loop back to check for letters to the editor, retractions and other corrections. Otherwise, they may be markedly misled to think that many more treatments have been shown to be effective than are truly effective. This is a taint on the obesity and nutrition literature, and we all need to pull together to do better with respect to the design, analysis and reporting of cluster randomized trials so that research study funds, research participants' time, and all other resources that go into these studies are not wasted, and our field is not misled. For a fuller discussion on the types of errors made in design, analysis and interpretation of this type of study, please refer to the references cited above.

### (c) Non-randomized designs and analyses for them that are stronger than standard non-randomized studies

Conventional epidemiological studies are subject to confounding by either upstream factors such as smoking, which influences both body mass index (BMI) and health outcomes, or when early stages of the disease process influence both BMI and mortality risk (often referred to as reverse causality). These processes will generally lead to apparent elevation of mortality risk at low adiposity levels and attenuation of the positive association between BMI and mortality at higher levels. Together these will lead to the conventional observational associations being entirely misleading if they are taken to be indicators of the shape of causal effects. Various approaches have been introduced to obtain more reliable causal estimates from observational data [44–47]. The underlying principle is not that one approach is always superior for obtaining reliable causal evidence on adiposity and health outcomes. Instead, a range of approaches—each of which may be biased, but if so hopefully by different factors, to different extents and in different directions—are considered within a triangulation of evidence framework.

#### (i) Co-twin and sibling control studies

Confounding can be reduced in epidemiological studies through family designs. The co-twin control design investigates how the difference in exposure level between monozygotic twins relates to the difference in the outcome. It is clear that this design controls for potential confounding by germline genetic variants and by environmental factors shared by children raised together (sometimes referred to as shared environment) [48]. Thus, within monozygotic twin pairs discordant for BMI, the influence of BMI on downstream traits has been used to estimate the effects of adiposity on an array of molecular phenotypes plausibly influenced by adiposity [49]. While the approach can reduce the range of potentially confounding variables, it is not robust to reverse causation (for more on reverse causation see [50]); if one twin is developing a disease and this process leads to falling BMI, then this will seriously influence the within-twin pair analysis of the association between BMI and mortality, for example. Indeed, by removing other sources of variation within monozygotic twin pairs, such studies could exacerbate the magnitude of confounding due to reverse causation. This  has been termed ‘Z-bias' in the causal inference literature and occurs more generally when adjusting for an instrumental variable has the unintended bias-amplification effect [51,52].

#### (ii) Mendelian randomization

Mendelian randomization uses the unique properties of germline genetic variants that are supported by the laws of Mendelian inheritance [53]. The paradigmatic form, outlined in the introduction of the approach, is of a between-sibling study, in which genetic variants are randomized from parents to their offspring [53]. Many enhancements to Mendelian randomization have also been offered (e.g. [54–56]). If genetic variants are reliably associated with an in-principle modifiable phenotype, then their association with an outcome influenced by that phenotype allows estimation of the causal effect of the modifiable risk factor on the disease outcome [55,56].

Mendelian randomization is generally implemented within an instrumental variables (IV) framework, for which three assumptions are necessary: (1) the genetic variant is robustly associated with the exposure of interest; (2) there is no confounding between the genetic variant and the outcome; (3) the genetic variant does not influence the outcome except through the exposure phenotype under investigation [56]. There are many ways in which these assumptions can be violated, and Mendelian randomization has resulted in the development of a broader range and more stringent set of sensitivity analyses than previously existed in the IV field [56].

#### (iii) First-degree relative measures as an instrumental variable

Related to Mendelian randomization, it is also possible to use first-degree relative phenotypic measures, for example, of BMI, as IVs for the index individual. Thus, the BMI of offspring can be used as an instrumental variable for the BMI of their parents. Reverse causation—where early stages of a disease process lead to loss of weight—is a major confounder in studies of BMI and mortality. However, parental illness will not generally lead to a change in the BMI of their adult offspring. This approach has been used in many studies, with the general finding that a steeper positive association, with no upturn at low BMI, is seen between BMI and all-cause mortality [57]. Combining offspring BMI and own germline genetic variation as instruments is a powerful technique for causal effect estimation [58].

#### (iv) Cross-context comparisons

The confounding contribution to associations seen in observational studies can be explored by deliberately selecting samples from contexts in which the confounding structure will differ. For example, consider investigating the influence of being breastfed on later obesity. In high-income countries, many studies suggest that breastfeeding reduces the prevalence of obesity in offspring [59]. However, breastfeeding is strongly socially patterned, and confounding would generate associations in the same direction across all studies. If, however, populations are chosen for which the confounding structure is known to differ substantially, the findings generated by confounding would be anticipated to vary widely between them, but causal effects would not differ between the populations. Comparing data from a high-income country cohort, in which breastfeeding was strongly socially patterned, with those from a middle-income country cohort in which there was no marked social patterning of breastfeeding is one example of this design. Associations in opposing directions were seen, with breastfeeding apparently protecting against later obesity in the high-income country but not in the middle-income country cohort. The finding of a lack of effect of breastfeeding on later obesity has also been seen in a large cluster randomized controlled trial [60].

#### (v) Negative controls

Causal inference can be strengthened through the incorporation of negative and positive controls. A negative control exposure cannot plausibly influence the outcome under investigation but would be subject to similar confounding as the exposure of interest. For example, when investigating the intrauterine effect of maternal smoking during pregnancy on offspring birthweight and offspring obesity, an apparent negative control exposure would be that of paternal smoking, which would not plausibly influence the intrauterine environment [44,61,62] (the biological effects of passive smoking through maternal absorption in these circumstances would be tiny compared with that of own maternal smoking behaviour). Maternal smoking strongly relates to offspring birth weight, but the negative control—paternal smoking—is unrelated to offspring birth weight.  This strengthens evidence regarding the causal effect of maternal smoking in the birthweight case.  For offspring obesity, both maternal and paternal smoking relate in similar ways, casting doubt on their being a causal effect of intrauterine exposure to maternal smoking on later obesity [44,45,61,62].

#### (vi) Packet randomized experiments

Packet randomized designs are not new. (Pavela and colleagues coined the name ‘packet randomized design’ in 2015 [63].) Rather, this class of designs has existed for a long time and has been widely used, including in obesity-related research. However, the common underlying principles, common advantages and common limitations of this class of designs have yet to be widely recognized.

In a packet randomized design, one recognizes that in some situations, units of observation (e.g. mice, rats, people) may be randomized to exposure to some other unit. In many cases, each experimental or research unit (the subject) may be exposed to a unique instance of exposure. This instance could be another mouse in a parabiosis experiment, another person in the form of a roommate to whom a freshman at college is randomly assigned, a person posing as a job interviewee to determine the effects of perceived obesity on hiring decisions, or a household to which adoptees are randomly assigned. We refer to the exposure set to which an experimental unit is assigned as the ‘packet’. This is because the packet contains within it some element that the experimenter is interested in, and by randomizing subjects to packets, the experimenter has successfully used randomization to break any dependency or correlation between the characteristics of the subject and the characteristics of the packet. This eliminates all confounding subject characteristics. However, the subject is not randomized just to different levels of the independent variable of interest (e.g. the value of some constituent of the blood of mice and parabiosis, BMI of a roommate, socioeconomic status of a household, etc.) but, in fact, is randomized to the entire exposure unit or packet, which comes with other characteristics that may be correlated with, or not independent of, the particular independent variable of interest.

As a result of the randomization, packet randomized experiments eliminate entire classes of confounders, specifically all those confounders that are due to the subject and their history, but not confounders inherent within the packet. For example, suppose roommates are assigned to each other at random, and individuals assigned to roommates (packets) who have higher BMIs gain less weight during their freshman year than do subjects assigned to roommates (packets) who have lower BMIs. In that case, we can eliminate as a competing explanation for this association any confounding by characteristics of the subject. That is, it is not, for example, that subjects with a tendency to gain less weight in their freshman year tend to choose heavier roommates or that subjects with a tendency to gain less weight in their freshman year tend to choose roommates with some other characteristic that is correlated with gaining less weight or that subjects whom roommates choose with higher BMIs tend to have genotypes that protect them from weight gain. All these confounders are eliminated. Yet, we cannot say with certainty that the roommate's BMI causes the co-roommate to gain less weight. It is possible that some other characteristic within the packet (roommate) that is associated with roommate BMI leads the subject's BMI to increase less during the freshman year. A fuller description of the packet randomized experimental examples (e.g. roommates) and explanation and suggestions for the design and analysis of such studies are contained in Pavela *et al*. [63].

#### (vii) Triangulation, generalization, and consilience

The concept of triangulation can be seen as sitting along a continuum from replication to generalization to triangulation to consilience. In replication, one tries to conduct a study that tests essentially the same hypothesis with a new dataset and perhaps slightly varied methods [64,65]. In generalization, one tests whether the same hypothesis seems to be supported (or equally unsupported) in a different population, under different circumstances, or with some more major difference in methods. In triangulation, one uses different methodological approaches that would not be biased by the same processes as each other. This means that if the findings point in the same direction stronger inference can be made about their causal bases. Conducting a mouse study with an injectable dose of the drug in question and a human study with oral consumption would be one example of such. Finally, consilience refers to a somewhat different concept in which one is not looking at multiple studies to assess whether the same hypothesis yields roughly the same or supportive results. Rather, we are asking whether the results and conclusions regarding a particular hypothesis either fit with, or are inconsistent with, current knowledge from cognate scientific disciplines [66]. So, for example, when findings or hypotheses seem not to accord with the laws of thermodynamics or require growth in stature among adults, they are suspect.

Triangulation of different types of evidence is increasingly used in biomedical and other research [44–46]. Triangulation embraces the 'variety of evidence' thesis that inferential strength depends not only on the quantity of available evidence but also on its variety: the greater the variety, the stronger the resulting support. An essential condition is that the systematic errors and biases are unrelated across different study types. For example, the effect of inhibiting HMGCoA reductase on the risk of coronary artery disease can be estimated from randomized controlled trials of statins or through Mendelian randomization using genetic variants related to HMGCoA activity. The processes generating results of both randomized controlled trials and Mendelian randomization studies could be biased. However, the potential biases in one study design would hopefully not influence estimates of the other approach: the biases are presumed to be unrelated. When biases are unrelated, it may be possible to obtain two or more estimates using different estimation strategies from the same single study sample; these have been referred to as ‘evidence factors’ [67], which can be meta-analysed to increase statistical power. An example is a study based on the UK Biobank of the effect of years of education on health and health behaviours [68,69]. Two different instrumental variables were used. The first was based on a quasi-experiment, the 1972 schooling reform in the United Kingdom, which raised the minimum school leaving age, and the second used Mendelian randomization. The two estimates could be meta-analysed in these circumstances [67]. Of course, how to formally or numerically combine disparate sources of information from animal studies, *in vitro* studies, human epidemiological studies, and so on is complex, and no one concrete procedure has been established, refined and accepted by the general scientific community. Such procedures were discussed (e.g. see §4f(iv) on ‘Borrowing Strength’) in a seminal National Academy of Sciences publication from 1992 [70] and later expanded on by the work of Bareinboim and Pearl [71].

#### (viii) The implication of non-manipulable factors in causal analyses

Pearl [72] explicitly takes on the question of non-manipulable factors in causal analyses in the context of obesity. He begins by noting that if we want, for example, to estimate the causal effect of obesity on mortality in humans we must wrangle with several issues, including especially that we can manipulate things that affect obesity, but cannot directly randomize humans to levels of obesity *per se*, a point that has been made previously in the obesity literature. For example, Yanovski *et al*. [73] wrote ‘Subjects in an RCT could not be randomly assigned to lose or not lose weight; they could only be randomly assigned to receive or not receive interventions that might result in weight loss. These interventions, however, might well produce changes in health status that are not due to weight loss. Promotion and maintenance of weight loss through increased physical activity, reduced saturated fat intake, and consumption of large amounts of fruit and vegetables are examples of such interventions. It may appear that one could never infer that weight loss itself caused the changes in health status. However, if participants in an RCT were randomly assigned to several interventions that produce weight loss through different mechanisms and these interventions yielded similar improvements in health status, then the conclusion that weight loss was responsible for the improvements in health outcomes may be justified’. Pearl [72] writes ‘There is of course a fundamental difference between smoking and obesity; randomization is physically feasible in the case of smoking (say, in North Korea)—not in the case of obesity. Yet it is not entirely unimaginable. An RCT on obesity requires more creative imagination, invoking not a powerful dictator, but an agent such as Lady Nature herself, who can increase obesity in a well-specified way (say 1% increase in fat content of a certain muscle) and evaluate its consequences on various body functions. This is what the do-operator does, it simulates an experiment conducted by Lady Nature who, for all that we know is almighty, and can permit all the organisms that are affected by a given change (say in fat content) to respond to that change in the same way that they responded in the past. Moreover, she is able to do it by an extremely delicate surgery, without touching those variables that we mortals need to change in order to drive BMI up or down’.

#### (ix) Empirical *p*-value calibration

One of the concerns about causal inferences from observational data is that multiple factors may lead to *p*-values not accurately reflecting the probability of observing data that depart as much or more extremely under the null hypothesis and with all assumptions met as do the observed data, i.e. the definition of *p*-value. This could occur if one considers the null hypothesis to be one of causation and not merely association, a result of residual confounding, or other artefacts such as measurement errors, departures from statistical model assumptions, or non-independence of residuals.

To address this, Schuemie *et al*. [74] introduced the idea of empirical *p*-value calibration, which can be seen as an extension of the concept of negative controls. Here, the negative control is an alternative exposure variable. In brief, one conducts an analysis in which many exposure variables, most if not all of which are thought to be implausibly causal with respect to their effects on the outcome, are run through the same analysis as the exposure variable of actual interest.

If any of these turn out to be statistically significant, then the significance of any association with the exposure variable of interest is called into question. More formally, one can then create a calibration curve to recalibrate the observed *p*-value for the main hypothesis test of interest and adjust it as described in these articles [74,75]. Whether these *p*-values should be called *p*-values or something else is open to question, but the concept holds.

Nevertheless, the method has also been criticized elsewhere [76,77]. The method is not a panacea but can be one additional method in the armamentarium of investigators trying to draw causal inferences from observational association data with the greatest rigour.

### (d) Recommendations on causal effect interpretation

The following discussion will focus on experimental studies and assume that study participants were properly randomized for the intervention.

#### (i) Average versus individual causal effects

The idea of 'potential outcome' is commonly used in various statistical causality methods. It can be viewed as a foundational and complementary concept [22] or as a derived concept such as in the Structural Causal Model of Pearl [4]. In its simplest form, with a binary *causal variable* X and *response variable* Y, we introduce, for each individual, a pair of variables, Y_0_ and Y_1,_ where Y_x_ denotes the value Y would take for that individual were X to be externally set to take value x. The *individual causal effect* of X on Y is then defined as ICE = Y_1_ – Y_0_. Note, however, that because it is not possible simultaneously to set X both to 1 and to 0, we can never observe both Y_1_ and Y_0_ for the same individual, so we can never observe ICE. This has been termed ‘the fundamental problem of causal inference’ [78].

A different approach considers not a value, but a probability distribution, P_x,_ for Y, after setting X to x. We define the *average causal effect*, ACE, as E_1_(Y) – E_0_(Y), where E_x_ denotes expectation under distribution P_x_ (so E_x_(Y) = P_x_(Y = 1)). Using data from a randomized experiment, we can estimate P_1_ (resp, P_0_) from responses of those assigned to treatment, X = 1 (respectively, those assigned to control, X = 0), and so estimate ACE. Here there is no fundamental problem.

Reverting to ICE, we note that E(ICE) = E(Y_1_) – E(Y_0_) — and, because E(Y_x_) = E_x_(Y), we can estimate each term, as above. So even though ICE is not observable, we can estimate its expectation, which is again ACE. Attention is now diverted from ICE to ACE. It has been argued [23] that consideration of the non-identifiable pair of potential outcomes Y_x_ buys us nothing that cannot be expressed more straightforwardly, starting with just the pair of estimable distributions P_x_.

Where potential outcomes do seem essential, however, is in attributing causation or blame. Suppose an individual has known values, say X = 1 and Y = 1. We ask, ‘Did the setting of X to 1 *cause* the response Y = 1?’ One interpretation of this question is: ‘Would Y have been different if X had been different?’ This is addressed by the probability of causation (PC): the probability of Y_0_ = 0, in light of the available information X = 1, Y_1_ = 1. For this, we must consider a joint probability distribution for Y_0_ and Y_1._ Because of the fundamental problem of causal inference, this, and hence PC, will typically be inestimable from data. However, it is possible to use P_0_ and P_1_ to set bounds on PC [24,79–82]^6^.

#### (ii) Effect size interpretation

A useful effect size metric when considering the value of interventions, particularly in the community and public health domain, is the common language effect size (CLES) indicator. The CLES ‘expresses how often a score sampled from one distribution will be greater than a score sampled from another distribution’ [84]. We have found it useful to put into perspective the magnitude of effects so that a non-scientist might appreciate them and use them in administrative or policy-level decision-making [85].

Next, when considering variances, it is important to note that there is no single ‘the variance’. One might be interested in the variance in the outcome among individuals or the variance in the mean outcome among individuals in clusters such as schools, clinics, counties, platoons, etc. One might be interested in the total variance in a trait without conditioning on any factors other than the inclusion criteria for the study or, conversely, the residual variance in a trait after conditioning on some covariates. One might be interested in the variance in the implicit population defined by the inclusion and exclusion criteria in a study or in some broader population. One might be interested in the variance of the measured outcome or of the latent variable that the measured outcome (which presumably includes measurement error) is intended to represent. Each of these requires different calculations, which meta-analysts and other investigators often ignore. A thorough discussion can be found in [86]. One particular area in which there is much confusion involves cluster randomized trials. See for example [87,88] for a specific case discussion. For a more general case, see [89], and beyond the obesity literature, see [90].

The consideration of what metrics are appropriate for estimating causal effects will depend on the goal of the communication. That is, the statistics should be ‘fit for purpose’. It is especially noteworthy that what constitutes an important, large, or valuable effect size is partly subjective and partly involves the perspective of the estimator or user of the statistic. For example, in a paper discussing the potential value of personalized prediction of the outcomes of bariatric surgery, we showed that an effect size that might seem trivial from an individual patient's perspective could be profoundly valuable from the payer's perspective where the number of people who could benefit from bariatric surgery exceeds the amount of money available to pay for the bariatric surgeries [91].

Similarly, the value of any given magnitude of effect size may depend on whether one is mainly hoping to predict outcomes, identify targets for further study (especially molecular targets for drug discovery), or exploit a causal effect for achieving some goals, such as treating or preventing obesity. We should not conflate these. Seemingly small effects may at times be important and we should value them; conversely, seemingly large effects may not be of interest in other contexts.

#### (iii) Effect under proposed conditions of use

In most circumstances involving the causal effects of treatments proposed for use in humans, we wish to know the effects under the proposed actual use conditions. The enjoinder to test for effects under the proposed conditions of use is codified in, for example, certain FDA documents. At the same time, we wish to know the effects of the treatment (e.g. drug) above and beyond any expectancy, placebo, or other social or cognitive factors that emanate from the knowledge that one is taking the treatment. This latter effect (the 'placebo-corrected effect') is estimated by running blinded randomized controlled trials. And yet, the proposed conditions of use rarely, if ever, involve blinding. That is, we do not propose, for example, that physicians provide drugs to their patients but tell the patient that they may be receiving a placebo, and that neither the physician nor the patient will know whether a placebo or active drug has been dispensed. So, by estimating the effects of the treatment (e.g. drug) above and beyond any expectancy, placebo, or other social or cognitive factors that emanate from the knowledge that one is taking the treatment, one is inherently NOT testing for the effects under proposed conditions of use. Can this quandary be overcome? Perhaps. George *et al*. [40] ‘propose an experimental design, Randomization to Randomization Probabilities (R2R), which significantly improves estimates of treatment effects under actual conditions of use by manipulating participant expectations about receiving treatment. In the R2R design, participants are first randomized to a value, π, denoting their probability of receiving treatment (versus placebo). Subjects are then told their value of π and randomized to either treatment or placebo with probabilities π and 1 – π, respectively. Analysis of the treatment effect includes statistical controls for π (necessary for causal inference) and typically a π-by-treatment interaction. Random assignment of subjects to π and disclosure of its value to subjects manipulates subject expectations about receiving the treatment without deception’. Other ways to accomplish this involve deception [40], but that is ethically objectionable, especially for long-term treatment studies. Therefore, the R2R design permits superior estimation of the causal effects of treatments for actual clinical use. It also affords interesting possibilities for estimating how actual treatment effects (not just placebo or expectancy effects) may vary as a function of expectations (i.e. expectancy by actual treatment interaction effects).

##### Moderation by preference and choice

There are likely marked differences among individuals in terms of how they respond to treatments. However, 'very likely' or 'very plausible' are different from what is demonstrated. We need more designs in the future that use multiple points of randomization to better estimate individual treatment effects and treatment response heterogeneity variance.

Regarding choice and preference, again, one cannot, without lying, randomly assign people to treatment conditions and at the same time have them choose their treatment conditions. Therefore, the conventional designs estimate the average response to treatment among people who are not choosing their treatment. By contrast, it seems plausible that the effects of receiving treatment might depend in part on whether one has voluntarily chosen that treatment. How can this be addressed? There is no simple answer, but one can construct designs in which, for example, one first randomizes individuals either to be randomized or to have their preferred treatment. Those participants randomized to ‘choose’ are allowed to select their preferred treatment, whereas participants assigned to be randomized are randomly assigned to treatment conditions. One can then test for interactions between the mode of treatment assignment and the treatment assigned on the outcome. If no interaction is observed, this suggests (but does not prove) that the effects of receiving the treatment do not depend on whether one voluntarily chooses that treatment. By contrast, if there is an interaction between the mode of treatment assignment and treatment assigned, this suggests (but does not prove because of the non-randomized nature of one aspect of the design) that the effects of receiving or being assigned to a particular treatment depend on whether one voluntarily chose that treatment.

The above-mentioned design is similar to but slightly different from asking whether the effects of treatment depend on one's preferred treatment. For example, one could ask a group of participants whether they prefer treatment A or treatment B (e.g. a low-fat diet or a low-carbohydrate diet). One could then randomly assign all the participants to receive one of the two treatments. This would be an ordinary randomized control parallel design. However, because preference would have been recorded before the study was initiated, one can then ask whether treatment preference interacts with treatment assignment in terms of the apparent effect on the outcome. Again, if no significant interaction is observed, one tentatively concludes that the effect of treatment assignment is not associated with preference. Indeed, that is the most common finding of such studies [92,93]. We have discussed in a previous paper [38,94] a number of studies that do not demonstrate an interaction between receiving one's preferred treatment and treatment assignment on weight loss outcome. By contrast, if one observes a statistically significant interaction between treatment preference and assigned treatment, then one can tentatively state (but not state that it has been proven, because of the non-randomized assignment to preference) that the effect of receiving treatment A versus treatment B is associated with whether one prefers treatment A or treatment B (presence of effect heterogeneity). However, we do not know if the effect heterogeneity is due to the treatment preference or something else associated with it. Though it seems intuitively reasonable that this would be common, as mentioned earlier, this seems to be something other than what the data most often support.

#### (iv) Effect when there is interference (spillover)

When individuals receiving the intervention cannot be considered totally isolated from the rest of the population, Rubin's stable unit treatment value assumption (SUTVA) [95] cannot reasonably be thought to hold in the estimation of a treatment's causal effect. In such a case, we can suspect that ‘spillover’ or ‘interference’ exists. Ignoring the spillover effect can lead to a biased estimate of the true causal treatment effect. Hudgens and Halloran [96] were the first to propose causal estimands of the spillover effect in the two-stage randomized setting. When the group sizes are different, estimation of the causal estimands presented in Hudgens and Halloran [96] can be biased. Recently, Basse and Feller [97] proposed weighted (sample size weighted) estimates appropriate in non-equal group size setting. Recently, Tchetgen Tchetgen [98] proposed an approach to perform causal inference in a single realisation of network data.

As in most interventional studies, the effect of spillover is also an important issue in obesity research since behavioural changes (i.e. dietary intake or healthier eating) often lead to similar changes beyond one individual in a group [99,100]. Spillover in obesity research has also been thought of as the cascading effect that changes in one lifestyle factor (e.g. healthier eating) exert on other lifestyle factors (e.g. physical activity, alcohol consumption, etc.) See [101] for an in-depth description of the framework and identification of spillover effect in a healthcare interventions.

#### (v) Effect when there is contamination

The concept of contamination can be defined as the scenario where the participant in the control group received elements of the treatment [102]. Some definitions or usages of contamination are closely related to the concept of interference as defined above. However, other definitions imply similar but distinct concepts, including some that are closer to the idea of lack of treatment integrity or treatment fidelity [103,104]. We will not dwell on these concepts at length here but refer the reader to publications involving the assessment and interpretation of effect estimates when contamination is present or suspected, methods to minimize contamination, and the effect of contamination on effect estimates [96,105].

#### (vi) Treatment response heterogeneity

Here, we are interested in the idea of treatment response heterogeneity [106]. Treatment response heterogeneity seems to be commonly assumed to exist. Clinical investigators frequently comment on the apparent variability in responses among participants in trials. One often hears phrases like ‘responders’, ‘strong responders’ and ‘non-responders’. Yet, as many have pointed out [107–109], such statements are mostly articles of faith.

This is because treatment response heterogeneity has only actually been demonstrated in few cases. What has been demonstrated is variability in outcome, not necessarily variability in response. If variability in outcome were the same as variability in response, or more succinctly, if the response to treatment were the same as the outcome after receiving treatment, then we would not need controlled trials. We use controlled trials (whether placebo-controlled or not) to quantify the average effect of treatment or the average response to treatment by comparing the average outcome of the treatment group with the average outcome of the control or alternative assignment group. But the conventional design contains no information, strictly speaking, that would allow precise identification of individual treatment effects or of the variance in individual treatment effects. Treatment response heterogeneity between two subgroups does not imply that the subgroup factor causes the difference, given that the subgroup labels are not randomly assigned, and this has been discussed in greater detail in VanderWeele and Knol [110]. The dialogue around precision medicine and personalized nutrition seem to imply that greater variances observed in treatment response, beyond variation attributable to race, sex etc. can be explained by leveraging multi-omics data (Weathers & Gilbert [111]; Jayachandran *et al*. [112]; de Hoogh *et al*. [113]; National Academies of Sciences, Engineering, and Medicine [114]. However, several writings have argued that this is an unproven premise and that it is, in most cases, not demonstrated [115–120].

The variance can be bounded by mathematical techniques, as shown elsewhere [121,122]. To precisely estimate them and make them fully identifiable requires specialized designs. These are designs in which there are multiple randomization events for each participant, and each participant is exposed to each treatment condition more than once. Such designs have been described extensively by Senn [123] and Loop *et al*. [124]. These are similar to the conventionally known crossover designs but involve more than one randomization point. The conventional crossover design, especially with two periods, merely randomizes individuals to one of two sequences of treatments. Thus, there is only one randomization event, and sequence, time, and the individual person effect are not separately identifiable. That is, they are confounded.

## 6. Tolerance and expectation for multi-element inelegant models

In science, there is both deductive reasoning about cause and inductive reasoning. The latter generally refers to drawing inferences from empirical observations. A problem with induction is, as the philosophers of science say, that the data underdetermine the hypotheses or theories. In other words, for any given set of data, there can be more than one hypothesis or theory that may be consistent with the data. Therefore, it is generally accepted that no single explanation can be unequivocally proven to be the only explanation consistent with a set of evidence. How, then, does one decide among competing explanations? In the long run, one tries to prove some of them, or as Feynman & Sackett [1] put it, oneself, wrong. This is very much in the spirit of Popper's falsificationism, although most modern philosophers of science no longer find Popper's framework useful or *de rigueur*. However, until some explanations can be ruled out, competing explanations can be compared in numerous ways.

One way to compare competing explanations involves the law of parsimony, often phrased as Occam's razor. It is the spirit of the simplest explanation for a set of phenomena that explains it equally well being the best explanation that underlies the use of things. An example in statistics is Akaike's [125] information criteria, in which the degree of model fit is scaled relative to the number of parameters required to obtain that fit. The more parameters that are required to obtain an equally good fit, the more ‘fudge factors’ or fewer degrees of freedom one has, and the less compelling the evidence. As Johnny von Neumann is purported to have said, ‘with four parameters, I can fit an elephant, and with five, I can make him wiggle his trunk’ [126].

The appeal of parsimony has led many to search for elegant and simple theories, which are often perceived as beautiful. Beauty has its own value. Yet, as Strevens [127] and others have pointed out, there is no logical reason that a theory must be beautiful or why a more beautiful or parsimonious theory is necessarily the correct one. Indeed, in the field of physics, there is now a concern that the search for beauty in mathematical theories is leading the field astray. In some cases, it may seem true in biology as well.

In the field of evolutionary biology, for example, what is sometimes referred to as the queen of questions is why so many species reproduce sexually when by the usual math of it, asexual reproduction is far more evolutionarily advantageous to the reproducing organism. If one better transmits one's ‘selfish genes’ in the sense of Dawkins [128] through asexual reproduction, why engage in sexual reproduction? Many explanations have been put forth, and yet for almost none of them does the math really seem to work to overcome the disadvantage in genetic fitness that accrues from reproducing sexually versus asexually. To rescue this, we must give precedence to the observation that, in fact, sexual reproduction is extremely common and an adequate explanatory model is an ugly conglomeration of multiple simple and beautiful hypotheses. Each hypothesis on its own seems elegant and yet unable to explain the data. Altogether, these hypotheses may be sufficient, as described by Nick Lane, a fellow of the Royal Society, in his book *Life Ascending* [129]. In fact, this book received an award from the Royal Society, which is sponsoring this issue.

Similarly, in metabolic scaling, the 3/4-power law of metabolism has become the proposed universal law and, as Douglas Glazier discusses [130], many hypotheses have been generated over the past 80 years or more to explain this scaling. Nevertheless, there remains no consensus as to which model is most valid. Glazier argues that aspects of all these models, dependent on the contextual situation of the organism in question—for example, surface area-related fluxes of resources and wastes (including heat), internal resource transport, system composition and processes affecting resource demand—influence the level to which a model applies to that organism. While this may not lead to the ‘elegant model’, it does recognize and incorporate the complexity of life and the need for adaptation of organisms to their environments. Nick Lane makes similar arguments in his book *Power, Sex, Suicide: Mitochondria and the Meaning of Life* [131].

So too, perhaps it is time for us to stop thinking about *the* singular cause of obesity or *the* factor that led to the obesity pandemic, or the principal factor, and instead to be willing to embrace inelegant causal models that require the integration and conglomeration of multiple other factors. This may follow from the need for the constrained optimization inherent in multiple interconnected variables as opposed to simplistic ideas about the selection for or optimization of one single variable as though one could do so in a vacuum. On this latter point, consider the comments of Geary [132] in this issue.

## 7. Concluding remarks

In conclusion, the concept of causation is fundamental to understanding where the potential levers of action may lead to the ends we seek. Moreover, understanding of causal models and validated mechanisms has its own value in the experience of wonder and discovery. The consensus view in our field is that it is helpful to assume that causation exists and is, in principle, discoverable. However, too much research around obesity does not use the best design, measurement, or analytic and interpretive techniques to test and estimate causal effects optimally and to report on what we know separately from what we conjecture in straightforward, objective, transparent and honest ways. There is much room to improve, and a substantially enhanced toolbox to help is now available. Let us use it.

## Data Availability

This article has no additional data.

## Footnotes

1 We use ‘parameter’ as Altman and Bland (https://www.ncbi.nlm.nih.gov/pmc/articles/PMC1116021/) define the term to refer to aspects of a population distribution or model, and not to random variables (i.e. measures) that are observed in the study.

2 https://tinyurl.com/3c2f9ycc.

3 There is disagreement Dawid [23] as to whether this model term relates to a genuine real-world quantity, and in particular whether a joint distribution for (Y_0_, Y_1_) has any real-world meaning. We note that the joint distribution for (Y_0_, Y_1_) is not needed in this paper.

4 We will use the words confounding, confounder, confound and related words herein without providing thorough and careful definition. We recognize that many investigators define these quite differently and defining them in clear and uniform ways has challenged the field. This is well described in many places, including by Pearl [26]. We refer the reader there for more details. Here, we speak of the idea somewhat loosely and leave rigorous discussions to other venues.

5 We also note that covariate adjustment and re-randomization can improve conditional bias and hence unconditional efficiency of the difference of means estimators. Additionally, what is often referred to as a *failed randomization* simply refers to chances of imbalance in the important covariates. This is about precision, not bias. See Stephen Senn's blog (https://errorstatistics.com/2020/04/20/s-senn-randomisation-is-not-about-balance-nor-about-homogeneity-but-about-randomness-guest-post/) for further discussion.

6 We direct readers interested in better understanding the difference between the causal graph framework of Pearl and the decision-theoric framework of Dawid to a publication by Pearl (Pearl, J. [81,83]).

## Appendix

1. Natural Experiments [133]
2. Quasi-Experiments [134]
